# Role of ^18^F FDG-PET-CT in Fever and Inflammation of Unknown Origin

**DOI:** 10.3390/jcm14165861

**Published:** 2025-08-19

**Authors:** Elena Romano Gargarella, Flaminia Vocaturo, Andrea Guarneri, Maria Lucia Calcagni, Lucia Leccisotti

**Affiliations:** 1 Section of Nuclear Medicine, Department of Radiological Sciences and Hematology, Università Cattolica del Sacro Cuore, 00168 Rome, Italy; flaminia.vocaturo01@icatt.it (F.V.); marialucia.calcagni@unicatt.it (M.L.C.); lucia.leccisotti@unicatt.it (L.L.); 2 Unit of Nuclear Medicine, Fondazione Policlinico Universitario A. Gemelli IRCCS, 00168 Rome, Italy; andrea.guarneri@guest.policlinicogemelli.it

**Keywords:** infection, inflammation, FDG, PET/CT, FUO, IUO, fever of unknown origin, inflammation of unknown origin

## Abstract

Fever of unknown origin (FUO) and inflammation of unknown origin (IUO) remain complex diagnostic challenges due to their heterogeneous presentations and broad differential diagnoses. FUO was first described by Petersdorf and Beeson in 1961 and later redefined by Durack and Street, while IUO was introduced more recently by Vanderschueren et al. in 2009. Despite thorough investigations, a significant proportion of patients remain without a clear diagnosis, often resulting in prolonged hospital stays and increased healthcare costs. In recent years, [^18^F]FDG PET/CT has emerged as a valuable tool in the diagnostic workup of FUO and IUO, offering both metabolic and anatomical insights in a single scan. This review evaluates the diagnostic utility of [^18^F]FDG PET/CT, based on an analysis of 55 studies encompassing 6681 patients. The scan was found to be clinically helpful in 59% of cases, with diagnostic contributions from both true-positive and true-negative findings. Negative scans were frequently associated with spontaneous symptom resolution and fewer unnecessary interventions. However, differences in study design and definitions of diagnostic value make it difficult to compare results across studies. Overall, [^18^F]FDG PET/CT has proven to be a useful tool in the evaluation of FUO and IUO, and future research should focus on standardizing how its clinical benefit is measured and directly comparing its effectiveness with conventional imaging in well-designed prospective studies.

## 1. Introduction

Fever of unknown origin (FUO) and inflammation of unknown origin (IUO) present significant diagnostic challenges in clinical practice.

FUO was first described by Dr. Petersdorf and Dr. Beesom in 1961 [1]; since then, the definition has undergone several revisions, with the latest widely accepted version proposed by Durack and Street in 1991, characterizing it as a fever of 101 °F (38.3 °C) or higher on more than three occasions, without a diagnosis despite 3 days of relevant inpatient workup or three outpatient visits [2].

IUO, on the other hand, has a more recent history, first described by Vanderschueren et al. in 2009, who outlined it as an illness lasting more than three weeks, during which the body temperature is ≤38.3 °C on several occasions, the inflammatory markers are elevated (C-reactive protein (CRP) ≥ 30 mg/L or increase erythrocyte sedimentation rate), and for which no diagnosis could be established, despite minimal investigation, during at least three outpatient visits or 3 days of hospital investigation [3].

FUO and IUO are diagnostically challenging syndromes with wide global variation in incidence and aetiology. FUO is estimated to account for 2–3% of hospital admissions in high-income countries [4], but causes vary significantly by geography and socioeconomic status. Infections remain the most common cause globally, especially in low- and middle-income regions where tuberculosis and enteric infections are prevalent [4,5]. A multicenter cohort across 21 countries revealed that infections accounted for 51.6% of FUO cases, with neoplasms in 11.4%, collagen-vascular (non-infectious inflammatory) disorders in 9.3%, miscellaneous causes in 7.7%, and 20.1% staying undiagnosed [6]. A separate meta-analysis encompassing more than 1600 FUO/IUO patients found that rheumatic diseases were responsible for 22.2% of cases, led by adult-onset Still’s disease (≈22.8%), giant cell arteritis (≈11.4%), and systemic lupus erythematosus (≈11.1%) [7]. Geographic variation is notable: infections predominate in low- and middle-income countries (43–63%), whereas in upper-middle and high-income settings, infections comprise 15–49%, and collagen-vascular causes rise to 19–47% [6]. FUO/IUO more commonly affects middle-aged and older adults, often with comorbidities like cancer or immunosuppression that increase diagnostic complexity. Undiagnosed cases, however, remain in up to 10–20% of series, even in advanced healthcare settings [4,6].

Despite thorough initial evaluations, many patients still remain undiagnosed, leading to prolonged workups, increased healthcare costs, and compromised outcomes. Conventional laboratory assays (e.g., CBC, ESR, CRP, blood cultures, serologies) are indispensable in FUO/IUO evaluation, yet they remain inherently nonspecific—elevated inflammatory markers cannot reliably differentiate between infection, malignancy, and autoinflammatory disorders—and routine cultures and serologies often yield no diagnosis without specific clinical clues [8]. Likewise, abdominal and chest ultrasound are low-cost and radiation-free but suffer from limited sensitivity for deep, small, or retroperitoneal lesions, operator dependence, poor acoustic windows (e.g., obesity, bowel gas), and no scout imaging to ensure full coverage [9,10]. Due to these gaps, up to ~40–50% of FUO/IUO cases remain undiagnosed after first-line testing, often necessitating advanced imaging such as ^18^F-FDG PET/CT, whole-body MRI, or invasive tissue biopsy to identify aetiology [9,11].

With a wide range of possible differential diagnoses, spanning from infectious to non-infectious diseases, from malignancies to miscellaneous disease, a systematic, multidisciplinary approach is essential to identify active disease early and to guide the right therapy.

In recent years, [^18^F]FDG PET/CT has become a key tool in the diagnostic workup for FUO and IUO. Its use in everyday clinical settings—either as a first- or second-line modality—is becoming increasingly common, particularly when conventional imaging fails to identify a cause. As set forth by the European Association of Nuclear Medicine (EANM), this imaging technique combines metabolic and anatomical information in a single scan, allowing for precise localization and characterization of pathological FDG uptake [12]. Moreover, recently published Delphi-generated consensus-based recommendations on FUO/IUO listed [^18^F]FDG PET/CT in the strong consensus agreement as “an important diagnostic test (to perform) after a patient fulfills the FUO criteria with minimal diagnostic tests” and that “clinicians should consider earlier use of [^18^F]FDG PET/CT, after plain radiography or CT, particularly in the absence of potential diagnostic clues” [8]. Recent studies, including work by Buchrits et al. [13], report a diagnostic contribution in over 75% of cases, highlighting its ability to both detect underlying disease and exclude focal pathology. As nuclear medicine advances, [^18^F]FDG PET/CT is expected to further enhance diagnostic accuracy. Its integration into the diagnostic pathway helps optimize outcomes, reduce unnecessary interventions, and improve the overall management of patients with FUO and IUO.

This review aims to assess the diagnostic performance of [^18^F]FDG PET/CT in the evaluation of FUO and IUO, as supported by the current literature.

## 2. Methodology

A comprehensive search of the existing literature was performed on PubMed and Cochrane library from 2001 up to May 2025, using the following search terms: (PET OR FDG OR fluorodeoxyglucose) AND (fever OR FUO OR PUO OR pyrexia OR inflammation OR IUO). We included prospective, retrospective, and ambispective studies that examined the contribution of [^18^F]FDG PET/CT to the investigation of “fever of unknown origin” as defined by Durack [2] and of “inflammation of unknown origin” as defined by Vanderschueren” [3]. The complete PubMed search string is presented in Table 1 [14,15,16,17,18,19,20,21,22,23,24,25,26,27,28,29,30,31,32,33,34,35,36,37,38,39,40,41,42,43,44,45,46,47,48,49,50,51,52,53,54,55,56,57,58,59,60,61,62,63,64,65,66,67,68].

We excluded studies that assessed the contribution of [^18^F]FDG PET alone without the component of CT and studies that assessed [^18^F]FDG PET associated with MRI.

The primary goal was to assess the clinical helpfulness of [^18^F]FDG PET/CT to the final diagnosis of fever of unknown origin or inflammation of unknown origin, which was defined, depending on the study, as true positive or as true positive summed to the true negative (as reported in Table 1). We also reported sensitivity and specificity, positive predictive value, and negative predictive value of [^18^F]FDG PET/CT, as shown in Table 2 [14,15,16,17,18,19,20,21,22,23,24,25,26,27,28,29,30,31,32,33,34,35,36,37,38,39,40,41,42,43,44,45,46,47,48,49,50,51,52,53,54,55,56,57,58,59,60,61,62,63,64,65,66,67,68]. These values were obtained from the results published in the individual studies, either directly as reported by each study, or derived from the data presented in the articles whenever possible (the latter were marked with an asterisk (*) in Table 2). When extraction was not feasible, values were indicated as NR (not reported). For extracting these data, we constructed tables of TP, TN, FP, and FN results per study, from which sensitivity, specificity, PPV, and NPV were calculated.

## 3. Results

The literature search yielded 605 potentially pertinent publications. Among them, 549 articles were found to be unrelated to the subject or were the wrong study type. Ultimately, we included 55 studies, with a total of 6681 patients. We listed in Table 1 the general features of each study: this review included mostly retrospective studies (78%), a few prospective studies (20%), and only one ambispective study. Of these, thirty-six studies focused on patients presenting with fever of unknown origin, three studies examined patients with inflammation of unknown origin, and sixteen studies comprised both these populations. Fifty-three studies out of the total fifty-five analysed reported the number of patients who received a final diagnosis: out of 6567 patients for whom the study reported a final diagnosis, the underlying cause of FUO or IUO was identified in 4917 of them (75%); in most of the remaining cases, fever resolved spontaneously.

A precise definition for clinical helpfulness of [^18^F]FDG PET/CT was not always reported in the analysed studies: it was specified, as a matter of fact, in only twenty-nine studies (53%), as shown in Table 2. Among these twenty-nine studies, in nineteen works (65%), the diagnostic contribution of [^18^F]FDG PET/CT scans was delineated as impactful exclusively when abnormal uptake was localized to a specific organ or tissue, and subsequent specific conventional diagnostic modalities confirmed a definitive diagnosis (true-positive scans, TP). Ten studies defined [^18^F]FDG PET/CT as a “valuable” test when the imaging either provided information that directly led to the final diagnosis (TP) or when it showed no focal FDG uptake (true negative, TN), consistent with the absence of disease in the final diagnosis. In Table 2, we also reported the main data reported from the considered studies: sensitivity ranged from 36% to 100%, with an average of 83%; specificity, on the other hand, ranged from 15% to 100%, with an average of 59%; PPV and NPV yielded similar results, ranging, respectively, from 51 to 100% and from 55 to 100%, with a mean valued of 78% for PPV and of 65% for NPV.

Overall, [^18^F]FDG PET/CT was found to be contributory to the diagnostic workup in 59% of all examined cases (it was considered as clinically helpful in a total of 3919 patients over the 6681 observed). However, considerable heterogeneity was observed across the individual studies. The reported clinical utility of PET/CT varied widely, ranging from as low as 16% in one study (Khan et al. [63]) to as high as 98–100% in others (Liu et al. [64], Rosenbaum et al. [25]). This substantial variation may be attributed to several factors. Primarily, discrepancies in the definition of “clinical helpfulness” likely played a key role. In some studies, utility was defined narrowly, referring only to PET/CT findings that correlated with histopathological confirmation (i.e., true positives). In others, the definition encompassed a broader range of outcomes, including imaging that led to changes in clinical management, negative results associated with spontaneous remission, or any combination of these. In certain cases, authors did not provide a clear definition of diagnostic yield at all. Additionally, differences in the study populations may have influenced the findings. Some investigations focused exclusively on patients with fever of unknown origin (FUO), others on those with inflammation of unknown origin (IUO), while some included both conditions. Variability in sample size across studies also contributed to the observed heterogeneity.

## 4. Discussion

The diagnostic evaluation of patients with FUO or IUO presents significant methodological challenges due to the heterogeneity of potential aetiologies, the absence of universally accepted reference standards, and the considerable proportion of patients who remain without a definitive diagnosis. Timely and accurate diagnosis significantly influences the therapeutic approach in patients with FUO. Early identification of the underlying aetiology can lead to the initiation of appropriate treatment, adjustment of ongoing therapies, or a complete change in the therapeutic regimen. Moreover, a precise diagnosis can guide targeted diagnostic interventions, both invasive and noninvasive—such as biopsy, drainage procedures, serological testing, and cultures of blood, urine, or tissues—with potential implications for cost-effectiveness and clinical efficiency [17]. [^18^F]FDG PET/CT plays a crucial role in the diagnostic workup of FUO by directing the clinician toward the most suitable and accessible biopsy site, thereby facilitating histological confirmation of the underlying disease. Additionally, [^18^F]FDG PET/CT contributes indirectly by helping to rule out numerous potential causes, effectively narrowing the differential diagnosis [45].

Most existing studies only take into consideration [^18^F]FDG PET/CT as a useful tool when it directly correlates to the final diagnosis (true positive), while true-negative studies are often underreported, despite their relevance in excluding significant pathology, predicting spontaneous clinical resolution, and potentially reducing unnecessary diagnostic procedures [16,23,31,41,47,69]. Moreover, negative PET/CT scans have been significantly associated with spontaneous remission [70]. While true-positive (TP) findings are essential for establishing a definitive diagnosis, true-negative (TN) results are equally valuable in excluding potential causes. In the context of FUO and IUO, previous studies have reported that the absence of pathological [^18^F]FDG PET/CT uptake on PET/CT is frequently associated with spontaneous resolution [35,69]. This finding has important clinical implications, as supported by Takeuchi et al.’s recent meta-analysis [70], demonstrating that patients with negative PET-CT results are significantly more likely to experience spontaneous resolution of symptoms compared to those with positive findings. These results emphasize the utility of a negative PET-CT not only as a diagnostic exclusion tool but also as a means to guide clinical management. Specifically, it may help avoid unnecessary invasive diagnostic procedures and limit the use of potentially inappropriate treatments, such as empirical antibiotic therapy [71].

However, a substantial proportion of FUO/IUO patients—ranging from 7% to 62% in the studies taken into consideration in our literature search—remained undiagnosed after [^18^F]FDG PET/CT. In light of these limitations, the more general concept of “clinical helpfulness”—defined as the proportion of scans that inform subsequent clinical decision-making—offers a more comprehensive assessment of [^18^F]FDG PET/CT’s value in this population with respect to more classical parameters like specificity and sensitivity [72,73,74]. The analysed studies reported an overall clinical helpfulness ranging from 16% to 100% (with a mean value of 60%), with higher values observed when both true-positive and true-negative results were considered: indeed, studies that counted PET/CT scans as helpful when they showed either true-positive or true-negative results reported an overall usefulness of 61% to 100%. In contrast, studies that only considered true-positive results showed a usefulness ranging from 16% to 85%.

A sizable prospective study [55] by Chen’s group, which examined 524 patients over the course of 5 years, found [^18^F]FDG PET/CT to be positive in 91% of patients (477/524; diffuse or focal high uptake of FDG in various organs and tissues), and the model demonstrated excellent discrimination for infection (AUC = 0.88), malignancy (AUC = 0.93), and NIID (AUC = 0.95). Notably, several studies included in this analysis only unacknowledged focal uptake as impactful, while excluding other “nonspecific” uptake from consideration (such as diffuse uptake in the spleen, bone marrow, or symmetrical lymphadenopathy) [16,21,35,42,43].

In conclusion, while the accuracy of [^18^F]FDG PET/CT in evaluating FUO/IUO can be affected by a range of factors—including patient complexity, definitions of FUO/IUO, timing, and variability in reference standards—most studies agree that it often provides useful information for clinical decision-making. A positive scan can be key to making a diagnosis, and sometimes it is even essential. Positive scans frequently contribute to, and occasionally are indispensable for, establishing a diagnosis. Conversely, negative scans can rule out focal disease and are associated with a high likelihood of spontaneous remission, thus serving as valuable prognostic tools. Moving forward, well-designed prospective studies are needed to further refine its diagnostic role in FUO/IUO workup.

## 5. Study Limitations

This literature review on the diagnostic value of [^18^F]FDG PET/CT in FUO/IUO is limited by several methodological issues, primarily the predominance of retrospective studies; these designs introduce selection and interpretation bias, potentially overestimating diagnostic accuracy. Definitions of FUO/IUO varied across studies, and no standardized diagnostic gold standard was applied, complicating comparison of results. Diagnostic workups prior to PET/CT were not protocolized; referring physicians and centres determined timing and prior investigations, leading to heterogeneity in pre-scan evaluation. Variability in the interpretation of PET/CT scans also introduced potential bias: some studies considered true-negative results diagnostically helpful, while others did not, creating inconsistencies in outcome assessment. Population heterogeneity and variability in final diagnostic confirmation methods (e.g., biopsy vs. clinical follow-up) further impacted diagnostic performance, particularly specificity. In many cases, additional diagnostic tools were required to verify PET/CT findings, introducing verification bias. These limitations highlight the need for prospective, standardized studies to more accurately assess [^18^F]FDG PET/CT utility in FUO/IUO.

## 6. Conclusions and Future Directions

FUO and IUO continue to present significant diagnostic challenges due to the heterogeneity of clinical presentations, a wide range of potential underlying aetiologies, and the lack of a standardized diagnostic approach. The existing literature mirrors this complexity, making it challenging to compare or combine study results. [^18^F]FDG PET/CT has shown a valuable role in this setting, with positive scans often helping to establish a diagnosis, while negative scans may also be clinically relevant by excluding localized disease and suggesting a better prognosis. For future research, the contribution of PET/CT should be defined as the sum of true positives and true negatives to improve accuracy and consistency. This review highlights significant methodological limitations, including retrospective designs, inconsistent FUO/IUO definitions, and non-standardized diagnostic workups, which may overestimate [^18^F]FDG PET/CT accuracy. Future standardized randomized controlled trials are needed to better define its diagnostic utility in this heterogeneous patient population and to determine the most effective first-line imaging modality in the evaluation of FUO/IUO.

## Figures and Tables

**Table 1:** Characteristics of included studies.

First Author	Year	Patients *n*.	Population	Study Type	Final Diagnosis
Jaruskova [14]	2006	94	FUO	R	NR
Bleeker-Rovers [15]	2006	70	FUO	P	0.50
Keidar [16]	2008	48	FUO	P	0.60
Balink [17]	2009	68	FUO	R	0.65
Federici [18]	2010	14	FUO/IUO	R	0.71
Ferda [19]	2010	48	FUO	R	0.92
Kei [20]	2010	12	FUO	R	0.58
Ergül [21]	2011	24	FUO	R	0.54
Kubota [22]	2011	81	FUO	R	0.75
Pelosi [23]	2011	24	FUO	R	0.71
Sheng [24]	2011	48	FUO	R	0.75
Rosenbaum [25]	2011	24	FUO	R	1.00
Becerra Nakayo [26]	2012	20	FUO	R	NR
Crouzet [27]	2012	79	FUO	R	0.77
Kim [28]	2012	48	FUO	R	0.85
Pedersen [29]	2012	22	FUO	R	0.60
Manohar [30]	2013	103	FUO	R	0.67
Balink [31]	2014	140	IUO	R	0.74
Buch-Olsen [32]	2014	57	FUO	R	0.91
Tokmak [33]	2014	25	FUO	R	0.92
Balink [34]	2015	498	FUO/IUO	R	0.66
Gafter-Gvili [35]	2015	112	FUO	R	0.74
Singh [36]	2015	47	FUO	P	0.53
Bouter [37]	2016	72	FUO/IUO	R	0.83
Pereira [38]	2016	76	FUO	R	0.93
Hung [39]	2017	58	FUO	P	0.79
Abdelrahman [40]	2018	27	FUO	P	0.92
Garcia-Vicente [41]	2018	67	FUO	R	0.88
Schönau [42]	2018	240	FUO/IUO	P	0.79
Wang [43]	2019	376	FUO/IUO	R	0.91
Wang [44]	2020	147	FUO/IUO	P	0.88
Georga [45]	2020	50	FUO	R	0.78
Zhu [46]	2020	89	FUO/IUO	R	0.74
Kubota [47]	2021	128	FUO	P	0.72
Letertre [48]	2021	44	FUO	R	0.70
MuldersManders [49]	2021	104	FUO/IUO	R	0.65
Bilici Salman [50]	2021	97	IUO	R	0.90
Mahajna [51]	2021	128	FUO	R	0.74
Das [52]	2021	43	FUO	R	0.74
Yadav [53]	2021	51	FUO	P	0.88
Buchritis [54]	2021	303	FUO	R	0.72
Chen [55]	2022	524	FUO	P	0.91
Chen [56]	2022	326	FUO/IUO	R	0.91
Holubar [57]	2022	317	IUO	R	0.72
Ogut [58]	2022	58	FUO/IUO	R	0.90
Ly [59]	2022	103	FUO/IUO	P	0.56
Weitzer [60]	2022	300	FUO/IUO	R	0.84
Becker KK [61]	2024	77	FUO/IUO	R	1.00
Fathala [62]	2024	105	FUO	R	1.00
Khan [63]	2024	573	FUO	A	0.38
Liu [64]	2024	40	FUO	R	1.00
Kobayashi [65]	2024	45	FUO/IUO	R	0.71
Koreli [66]	2025	30	FUO/IUO	R	0.50
Yu [67]	2025	284	FUO	R	0.69
Greuez [68]	2025	93	FUO/IUO	R	0.59

FUO: fever of unknown origin; IUO: inflammation of unknown origin; R: retrospective; P: prospective; A: ambispective; NR: not reported.

**Table 2:** FDG PET-CT contributory results in examined studies.

First Author	Clinical Helpfulness	Sensitivity	Specificity	PPV	NPV
Jaruskova [14]	0.36	NR	NR	NR	NR
Bleeker-Rovers [15]	0.33 (TP)	0.88	0.77	0.70	0.92
Keidar [16]	0.90 (TP + TN)	1.00	0.81	0.81	1.00
Balink [17]	0.56 (TP)	1.00	0.90	0.93	1.00
Federici * [18]	0.50 (TP)	0.70	0.75	0.88	0.5
Ferda [19]	0.77	0.97	0.75	NR	NR
Kei [20]	0.42 (TP)	NR	NR	NR	NR
Ergül [21]	0.63	0.92	0.45	0.63	1.0
Kubota [22]	0.54	0.81	0.75	NR	NR
Pelosi * [23]	0.87 (TP + TN)	0.50	0.50	0.85	0.85
Sheng [24]	0.67	0.89	0.33	0.80	0.50
Rosenbaum [25]	1.00 (TP + TN)	NR	NR	NR	NR
Becerra Nakayo [26]	0.55 (TP)	0.78	0.83	0.92	0.62
Crouzet [27]	0.19	0.98	0.87	NR	NR
Kim [28]	0.56	0.92	0.23	NR	NR
Pedersen * [29]	0.83	0.67	0.71	0.83	0.50
Manohar [30]	0.60	0.90	0.97	0.98	0.83
Balink [31]	0.51	0.94	0.83	0.93	0.77
Buch-Olsen [32]	0.75 (TP + TN)	NR	NR	NR	NR
Tokmak [33]	0.60 (TP)	0.94	0.80	NR	NR
Balink [34]	0.59 (TP)	0.89	0.89	0.94	0.80
Gafter-Gvili [35]	0.66	0.72	0.58	0.74	0.54
Singh [36]	0.38 (TP)	NR	NR	NR	NR
Bouter [37]	0.65 (TP)	0.81	0.86	NR	NR
Pereira [38]	0.61	0.77	0.31	0.61	0.50
Hung [39]	0.72	0.79	0.56	0.83	0.50
Abdelrahman [40]	0.85 (TP)	0.95	0.67	0.96	0.67
Garcia-Vicente [41]	0.52	0.84	0.31	NR	NR
Schönau [42]	0.57 (TP)	0.91	0.22	0.65	0.62
Wang [43]	0.90	NR	NR	NR	NR
Wang [44]	0.58	0.88	0.15	0.59	0.47
Georga [45]	0.72 (TP)	0.94	0.50	0.86	0.75
Zhu [46]	0.74 (TP + TN)	0.84	0.26	NR	NR
Kubota [47]	0.33 (TP)	0.45	0.40	0.67	NR
Letertre [48]	0.44	0.85	0.37	0.58	0.70
MuldersManders [49]	0.45	NR	NR	NR	NR
Bilici Salman [50]	0.61 (TP)	0.67	1.00	1.00	0.26
Mahajna [51]	0.48 (TP)	0.70	0.37	0.70	0.34
Das [52]	0.91 (TP + TN)	0.77	0.33	0.83	0.25
Yadav [53]	0.63	NR	NR	NR	NR
Buchritis [54]	0.26	0.89	0.81	NR	NR
Chen [55]	0.91	NR	NR	NR	NR
Chen [56]	0.96	NR	NR	NR	NR
Holubar [57]	0.75 (TP + TN)	0.84	0.62	0.77	0.72
Ogut [58]	0.72 (TP + TN)	0.88	0.37	0.79	0.55
Ly [59]	0.19 (TP)	0.36	0.81	NR	NR
Weitzer [60]	0.54 (TP)	0.80	0.90	NR	NR
Becker KK [61]	0.61 (TP + TN)	NR	NR	NR	NR
Fathala [62]	0.72 (TP + TN)	0.72	0.29	0.68	0.33
Khan [63]	0.16 (TP)	NR	NR	NR	NR
Liu [64]	0.98	0.93	0.62	0.83	0.80
Kobayashi [65]	0.64	0.91	0.38	0.78	0.62
Koreli [66]	0.50 (TP)	1.00	0.33	0.60	1.00
Yu [67]	0.48	0.79	0.61	0.76	0.63
Greuez [68]	0.31	NR	NR	NR	NR

PPV: positive predictive value; NPV: negative predictive value; TP: true positive; TN: true negative; NR: not reported; *: extracted data.

## Data Availability

No new data were created or analyzed in this study.

## Abbreviations

The following abbreviations are used in this manuscript:

FUO	Fever of unknown origin
IUO	Inflammation of unknown origin
FDG	Fluorodeoxyglucose
PET-CT	Positron emission tomography/computed tomography
PPV	positive predictive value
NPV	negative predictive value
NR	not reported
TP	True positive
TN	True negative
R	Retrospective
P	Prospective
A	Ambispective

## References

[1] Petersdorf R.G., Beeson P.B. (1961). Fever of unexplained origin: Report on 100 cases. Medicine.

[2] Durack D.T., Street A.C. (1991). Fever of unknown origin—Reexamined and redefined. Curr. Clin. Top. Infect. Dis..

[3] Vanderschueren S., Del Biondo E., Ruttens D., Van Boxelaer I., Wauters E., Knockaert D.D. (2009). Inflammation of unknown origin versus fever of unknown origin: Two of a kind. Eur. J. Intern. Med..

[4] Wright W.F., Yenokyan G., Simner P.J., Carroll K.C., Auwaerter P.G. (2022). Geographic Variation of Infectious Disease Diagnoses Among Patients With Fever of Unknown Origin: A Systematic Review and Meta-analysis. Open Forum Infect. Dis..

[5] Kang S., Zheng R. (2024). Distribution of the causes of fever of unknown origin in China, 2013–2022. J. Transl. Int. Med..

[6] Erdem H., Baymakova M., Alkan S., Letaief A., Yahia W.B., Dayyab F., Kolovani E., Grgic S., Cosentino F., Hasanoglu I. (2023). Classical fever of unknown origin in 21 countries with different economic development: An international ID-IRI study. Eur. J. Clin. Microbiol. Infect. Dis..

[7] Betrains A., Moreel L., De Langhe E., Blockmans D., Vanderschueren S. (2022). Rheumatic disorders among patients with fever of unknown origin: A systematic review and meta-analysis. Semin. Arthritis Rheum..

[8] Wright W.F., Stelmash L., Betrains A., Mulders-Manders C.M., Rovers C.P., Vanderschueren S., Auwaerter P.G., International Fever and Inflammation of Unknown Origin Research Working Group (2024). Recommendations for Updating Fever and Inflammation of Unknown Origin From a Modified Delphi Consensus Panel. Open Forum Infect. Dis..

[9] Betrains A., Moreel L., Mulders-Manders C.M., Auwaerter P.G., Torné-Cachot J., Weitzer F., Terasawa T., Ly K.H., Schönau V., Blockmans D. (2024). Comparison of diagnostic spectrum between inflammation of unknown origin and fever of unknown origin: A systematic review and meta-analysis. Eur. J. Intern. Med..

[10] Minamimoto R. (2022). Optimal use of the FDG-PET/CT in the diagnostic process of fever of unknown origin (FUO): A comprehensive review. Jpn. J. Radiol..

[11] Tavakoli A.A., Reichert M., Blank T., Dinter D., Weckbach S., Buchheidt D., Schoenberg S.O., Attenberger U. (2020). Findings in whole body MRI and conventional imaging in patients with fever of unknown origin-a retrospective study. BMC Med. Imaging.

[12] Hess S., Noriega-Álvarez E., Leccisotti L., Treglia G., Albano D., Roivainen A., Glaudemans A.W.J.M., Gheysens O. (2024). EANM consensus document on the use of [^18^F]FDG PET/CT in fever and inflammation of unknown origin. Eur. J. Nucl. Med. Mol. Imaging.

[13] Buchrits S., McNeil R., Avni T., Fredman D., Guz D., Gafter-Gvili A. (2024). The Contribution of 18F FDG PET-CT for the Investigation of Fever of Unknown Origin and Inflammation of Unknown Origin. Am. J. Med..

[14] Jaruskova M., Belohlavek O. (2006). Role of FDG-PET and PET/CT in the diagnosis of prolonged febrile states. Eur. J. Nucl. Med. Mol. Imaging.

[15] Bleeker-Rovers C.P., Vos F.J., de Kleijn E.M.H.A., Mudde A.H., Dofferhoff T.S.M., Richter C., Smilde T.J., Krabbe P.F.M., Oyen W.J.G., van der Meer J.W.M. (2007). A prospective multicenter study on fever of unknown origin: The yield of a structured diagnostic protocol. Medicine.

[16] Keidar Z., Gurman-Balbir A., Gaitini D., Israel O. (2008). Fever of unknown origin: The role of ^18^F-FDG PET/CT. J. Nucl. Med. Off. Publ. Soc. Nuclear Med..

[17] Balink H., Collins J., Bruyn G.A., Gemmel F. (2009). F-18 FDG PET/CT in the diagnosis of fever of unknown origin. Clin. Nucl. Med..

[18] Federici L., Blondet C., Imperiale A., Sibilia J., Pasquali J.L., Pflumio F., Goichot B., Blaison G., Weber J.C., Christmann D. (2010). Value of (18)F-FDG-PET/CT in patients with fever of unknown origin and unexplained prolonged inflammatory syndrome: A single centre analysis experience. Int. J. Clin. Pract..

[19] Ferda J., Ferdova E., Zahlava J., Matejovic M., Kreuzberg B. (2010). Fever of unknown origin: A value of (18)F-FDG-PET/CT with integrated full diagnostic isotropic CT imaging. Eur. J. Radiol..

[20] Kei P.L., Kok T.Y., Padhy A.K., Ng D.C., Goh A.S. (2010). [^18^F] FDG PET/CT in patients with fever of unknown origin: A local experience. Nucl. Med. Commun..

[21] Ergül N., Halac M., Cermik T.F., Ozaras R., Sager S., Onsel C., Uslu I. (2011). The Diagnostic Role of FDG PET/CT in Patients with Fever of Unknown Origin. Mol. Imaging Radionucl. Ther..

[22] Kubota K., Nakamoto Y., Tamaki N., Kanegae K., Fukuda H., Kaneda T., Kitajima K., Tateishi U., Morooka M., Ito K. (2011). FDG-PET for the diagnosis of fever of unknown origin: A Japanese multi-center study. Ann. Nucl. Med..

[23] Pelosi E., Skanjeti A., Penna D., Arena V. (2011). Role of integrated PET/CT with [^18^F]-FDG in the management of patients with fever of unknown origin: A single-centre experience. Radiol. Med..

[24] Sheng J.F., Sheng Z.K., Shen X.M., Bi S., Li J.J., Sheng G.P., Yu H.Y., Huang H.J., Liu J., Xiang D.R. (2011). Diagnostic value of fluorine-18 fluorodeoxyglucose positron emission tomography/computed tomography in patients with fever of unknown origin. Eur. J. Intern. Med..

[25] Rosenbaum J., Basu S., Beckerman S., Werner T., Torigian D.A., Alavi A. (2011). Evaluation of diagnostic performance of 18F-FDG-PET compared to CT in detecting potential causes of fever of unknown origin in an academic centre. Hell. J. Nucl. Med..

[26] Becerra Nakayo E.M., García Vicente A.M., Soriano Castrejón A.M., Mendoza Narváez J.A., Talavera Rubio M.P., Poblete García V.M., Cordero García J.M. (2012). Análisis de costo-efectividad en el diagnóstico de fiebre de origen desconocido y el papel de la ^18^F-FDG PET-TC: Propuesta de algoritmo diagnóstico [Analysis of cost-effectiveness in the diagnosis of fever of unknown origin and the role of ^18^F-FDG PET-CT: A proposal of diagnostic algorithm]. Rev. Esp. Med. Nucl. Imagen Mol..

[27] Crouzet J., Boudousq V., Lechiche C., Pouget J.P., Kotzki P.O., Collombier L., Lavigne J.P., Sotto A. (2012). Place of ^18^F-FDG-PET with computed tomography in the diagnostic algorithm of patients with fever of unknown origin. Eur. J. Clin. Microbiol. Infect. Dis..

[28] Kim Y.J., Kim S.I., Hong K.W., Kang M.W. (2012). Diagnostic value of 18F-FDG PET/CT in patients with fever of unknown origin. Intern. Med. J..

[29] Pedersen T.I., Roed C., Knudsen L.S., Loft A., Skinhoj P., Nielsen S.D. (2012). Fever of unknown origin: A retrospective study of 52 cases with evaluation of the diagnostic utility of FDG-PET/CT. Scand. J. Infect. Dis..

[30] Manohar K., Mittal B.R., Jain S., Sharma A., Kalra N., Bhattacharya A., Varma S. (2013). F-18 FDG-PET/CT in evaluation of patients with fever of unknown origin. Jpn. J. Radiol..

[31] Balink H., Bennink R.J., Veeger N.J., van Eck-Smit B.L., Verberne H.J. (2014). Diagnostic utility of (18)F-FDG PET/CT in inflammation of unknown origin. Clin. Nucl. Med..

[32] Buch-Olsen K.M., Andersen R.V., Hess S., Braad P.E., Schifter S. (2014). ^18^F-FDG-PET/CT in fever of unknown origin: Clinical value. Nucl. Med. Commun..

[33] Tokmak H., Ergonul O., Demirkol O., Cetiner M., Ferhanoglu B. (2014). Diagnostic contribution of ^18^F-FDG-PET/CT in fever of unknown origin. Int. J. Infect. Dis. IJID Off. Publ. Int. Soc. Infect. Dis..

[34] Balink H., Veeger N.J., Bennink R.J., Slart R.H., Holleman F., van Eck-Smit B.L., Verberne H.J. (2015). The predictive value of C-reactive protein and erythrocyte sedimentation rate for ^18^F-FDG PET/CT outcome in patients with fever and inflammation of unknown origin. Nucl. Med. Commun..

[35] Gafter-Gvili A., Raibman S., Grossman A., Avni T., Paul M., Leibovici L., Tadmor B., Groshar D., Bernstine H. (2015). [^18F^]FDG-PET/CT for the diagnosis of patients with fever of unknown origin. QJM.

[36] Singh N., Kumar R., Malhotra A., Bhalla A.S., Kumar U., Sood R. (2015). Diagnostic utility of fluorodeoxyglucose positron emission tomography/computed tomography in pyrexia of unknown origin. Indian. J. Nucl. Med. IJNM Off. J. Soc. Nucl. Med. India.

[37] Bouter C., Braune I., Meller B., Sahlmann C., Ritter C., Meller J. (2016). ^18^F-FDG-PET/CT in unexplained elevated inflammatory markers. Join. Entities Nukl..

[38] Pereira A.M., Husmann L., Sah B.R., Battegay E., Franzen D. (2016). Determinants of diagnostic performance of ^18^F-FDG PET/CT in patients with fever of unknown origin. Nucl. Med. Commun..

[39] Hung B.T., Wang P.W., Su Y.J., Huang W.C., Chang Y.H., Huang S.H., Chang C.C. (2017). The efficacy of ^18^F-FDG PET/CT and ^67^Ga SPECT/CT in diagnosing fever of unknown origin. Int. J. Infect. Dis..

[40] Abdelrahman S.F.E., El-nasr S.D., Gadalla S.I.S.E.H. (2018). Value of 18-F-FDG PET/CT in assessment of patients with fever of unknown origin. Egypt. J. Radiol. Nucl. Med..

[41] García-Vicente A.M., Tello-Galán M.J., Amo-Salas M., Ros-Izquierdo J., Jiménez-Londoño G.A., La Rosa Salas B., Prado-Serrano Pradas G., Pena-Pardo F.J., Soriano-Castrejón Á. (2018). Do clinical and laboratory variables have any impact on the diagnostic performance of 18F-FDG PET/CT in patients with fever of unknown origin?. Ann. Nucl. Med..

[42] Schönau V., Vogel K., Englbrecht M., Wacker J., Schmidt D., Manger B., Kuwert T., Schett G. (2018). The value of ^18^F-FDG-PET/CT in identifying the cause of fever of unknown origin (FUO) and inflammation of unknown origin (IUO): Data from a prospective study. Ann. Rheum. Dis..

[43] Wang Q., Li Y.M., Li Y., Hua F.C., Wang Q.S., Zhang X.L., Cheng C., Wu H., Yao Z.M., Zhang W.F. (2019). ^18^F-FDGPET/CT in fever of unknown origin and inflammation of unknown origin: A Chinese multi-center study. Eur. J. Nucl. Med. Mol. Imaging.

[44] Wang W.X., Cheng Z.T., Zhu J.L., Xing M.Y., Zheng C.F., Wang S.J., Xie N.N., XianYu Z.Q., Song J.X. (2020). Combined clinical parameters improve the diagnostic efficacy of ^18^F-FDG PET/CT in patients with fever of unknown origin (FUO) and inflammation of unknown origin (IUO): A prospective study in China. Int. J. Infect. Dis..

[45] Georga S., Exadaktylou P., Petrou I., Katsampoukas D., Mpalaris V., Moralidis E.I., Arvaniti K., Papastergiou C., Arsos G. (2020). Diagnostic Value of ^18^F-FDG-PET/CT in Patients with FUO. J. Clin. Med..

[46] Zhu W., Cao W., Zheng X., Li X., Li Y., Chen B., Zhang J. (2020). The diagnostic value of ^18^F-FDG PET/CT in identifying the causes of fever of unknown origin. Clin. Med..

[47] Kubota K., Tanaka N., Miyata Y., Ohtsu H., Nakahara T., Sakamoto S., Kudo T., Nishiyama Y., Tateishi U., Murakami K. (2021). Comparison of ^18^F-FDG PET/CT and ^67^Ga-SPECT for the diagnosis of fever of unknown origin: A multicenter prospective study in Japan. Ann. Nucl. Med..

[48] Letertre S., Fesler P., Zerkowski L., Picot M.C., Ribstein J., Guilpain P., Le Moing V., Mariano-Goulart D., Roubille C. (2021). Place of the ^18^F-FDG-PET/CT in the Diagnostic Workup in Patients with Classical Fever of Unknown Origin (FUO). J. Clin. Med..

[49] Mulders-Manders C.M., Kouijzer I.J., Janssen M.J., Oyen W.J., Simon A., Bleeker-Rovers C.P. (2021). Optimal use of [18F]FDG-PET/CT in patients with fever or inflammation of unknown origin. Q. J. Nucl. Med. Mol. Imaging..

[50] Bilici Salman R., Gülbahar Ateş S., Satiş H., Tufan A., Akdemir Ü.Ö., Yapar D., Ataş N., Güler A.A., Karadeniz H., Babaoglu H. (2021). Diagnostic Role of ^18^F-Fluorodeoxyglucose Positron Emission Tomography for the Evaluation of Patients With Inflammation of Unknown Origin. J. Clin. Rheumatol..

[51] Mahajna H., Vaknin K., Ben Shimol J., Watad A., Abu-Much A., Mahroum N., Shovman O., Shoenfeld Y., Amital H., Davidson T. (2021). The Utility of 18FDG-PET/CT in Diagnosing Fever of Unknown Origin: The Experience of a Large Tertiary Medical Center. Int. J. Environ. Res. Public. Health.

[52] Das S., Sathyendra S., Hephzibah J., Karuppusami R., Gunasekaran K., Shanthly N., Miraclin A., Iyadurai R. (2021). Utility of positron emission tomography-computed tomography in the evaluation of fever of unknown origin in a resource-limited tropical nation. World J. Nucl. Med..

[53] Yadav B.K., Pannu A.K., Kumar R., Rohilla M., Kumari S. (2021). Fever of unknown origin in older adults: A prospective observational study from North India. J. Assoc. Physicians India.

[54] Buchrits S., Gafter-Gvili A., Eynath Y., Bernstine H., Guz D., Avni T. (2021). The yield of F^18^ FDG PET-CT for the investigation of fever of unknown origin, compared with diagnostic CT. Eur. J. Intern. Med..

[55] Chen J., Xing M., Xu D., Xie N., Zhang W., Ruan Q., Song J. (2022). Diagnostic models for fever of unknown origin based on ^18^F-FDG PET/CT: A prospective study in China. EJNMMI Res..

[56] Chen J.C., Wang Q., Li Y., Zhao Y.Y., Gao P., Qiu L.H., Hao K.J., Li H.B., Yue M.G., Zhou Y.S. (2022). Current situation and cost-effectiveness of ^18^F-FDG PET/CT for the diagnosis of fever of unknown origin and inflammation of unknown origin: A single-center, large-sample study from China. Eur. J. Radiol..

[57] Holubar J., Broner J., Arnaud E., Hallé O., Mura T., Chambert B., Sotto A., Roubille C., Gaujoux-Viala C., Goulabchand R. (2022). Diagnostic performance of ^18^F-FDG-PET/CT in inflammation of unknown origin: A clinical series of 317 patients. J. Intern. Med..

[58] Öğüt T.S., Erbasan F., Terzioğlu M.E., Tazegul G., Yazısız V. (2022). The Diagnostic Value of Fluoro-18 fluorodeoxyglucose (F-18 FDG) PET/CT in fever or inflammation of unknown origin: A retrospective study at a Rheumatology Clinic. Cureus.

[59] Ly K.H., Costedoat-Chalumeau N., Liozon E., Dumonteil S., Ducroix J.P., Sailler L., Lidove O., Bienvenu B., Decaux O., Hatron P.Y. (2022). Diagnostic Value of 18F-FDG PET/CT vs. Chest-Abdomen-Pelvis CT Scan in Management of Patients with Fever of Unknown Origin, Inflammation of Unknown Origin or Episodic Fever of Unknown Origin: A Comparative Multicentre Prospective Study. J. Clin. Med..

[60] Weitzer F., Nazerani Hooshmand T., Pernthaler B., Sorantin E., Aigner R.M. (2022). Diagnostic value of F-18 FDG PET/CT in fever or inflammation of unknown origin in a large single-center retrospective study. Sci. Rep..

[61] Becker K.K., Søholm J., Hess S. (2024). The Diagnostic Yield of [^18^F]FDG-PET/CT in a Heterogeneous In-Patient Population with Suspected Infection or Inflammation Is Comparable to Findings in Patients with Classic Fever of Unknown Origin. Diagnostics.

[62] Fathala A., Benkuddah R., Almuhaideb A. (2024). Performance and value of ^18^F-FDG PET/CT in patients with fever of unknown origin. Biomed. Rep..

[63] Khan D., Phulia A., Kumar S., Sarswat S., Kv S., Sagar S. (2024). Role of ^18^F-FDG PET/CT for providing a targeted approach for etiology of PUO. Nucl. Med. Commun..

[64] Liu B., Ma R., Shum E., Hormiz M., Lee S.T., Poon A.M.T., Scott A.M. (2024). FDG-PET/CT for investigation of pyrexia of unknown origin: A cost of illness analysis. Eur. J. Nucl. Med. Mol. Imaging.

[65] Kobayashi T., Miyamori D., Ito M. (2024). Retrospective study on clinical value and optimal use of [^18^F] FDG PET/CT for inflammation of unknown origin in Japanese patients. Sci. Rep..

[66] Koreli U.Y., Torun E.S., Adaş M. (2025). Role of Positron Emission Tomography-Computed Tomography Scan in Reaching Definite Diagnosis in Patients With Fever of Unknown Origin and Inflammation of Unknown Origin in Rheumatology Outpatient Clinic. Open Access Rheumatol. Res. Rev..

[67] Yu X., Wang S., Du N., Zhao H., Chen H. (2025). Diagnostic efficacy and necessity of 18F-FDG PET/CT in fever of unknown origin: Insights from a retrospective cohort study. Front. Med..

[68] Greuez C., Lorenzo-Villalba N., Bessac D.M., Vogel T., Blondet C., Weber J.C., Kaltenbach G., Imperiale A., Andrès E. (2025). Interest of ^18^F-Fluorodeoxyglucose Positron Emission Tomography/Computed Tomography for Fever and Inflammatory Syndrome of Unknown Origin in Elderly Patients: A Retrospective Real-Life Single-Center Study from a University Referral Hospital. J. Clin. Med..

[69] Eynath Y., Halperin E., Buchrits S., Gafter-Gvili A., Bernstine H., Catalano O., Avni T. (2023). Predictors for spontaneous resolution of classical FUO in patients undergoing PET-CT. Intern. Emerg. Med..

[70] Takeuchi M., Nihashi T., Gafter-Gvili A., García-Gómez F.J., Andres E., Blockmans D., Iwata M., Terasawa T. (2018). Association of ^18^F-FDG PET or PET/CT results with spontaneous remission in classic fever of unknown origin: A systematic review and meta-analysis. Medicine.

[71] Mulders-Manders C., Simon A., Bleeker-Rovers C. (2015). Fever of unknown origin. Clin. Med..

[72] Hess S. (2020). FDG-PET/CT in Fever of Unknown Origin, Bacteremia, and Febrile Neutropenia. PET Clin..

[73] Wright W.F., Kandiah S., Brady R., Shulkin B.L., Palestro C.J., Jain S.K. (2024). Nuclear Medicine Imaging Tools in Fever of unknown origin (FUO): Time for a Revisit and Appropriate Use Criteria. Clin. Infect. Dis. Off. Publ. Infect. Dis. Soc. Am..

[74] van Rijsewijk N.D., IJpma F.F.A., Wouthuyzen-Bakker M., Glaudemans A.W.J.M. (2023). Molecular Imaging of Fever of Unknown Origin: An Update. Semin. Nucl. Med..

